# Phytoplasma Infection Blocks Starch Breakdown and Triggers Chloroplast Degradation, Leading to Premature Leaf Senescence, Sucrose Reallocation, and Spatiotemporal Redistribution of Phytohormones

**DOI:** 10.3390/ijms23031810

**Published:** 2022-02-05

**Authors:** Wei Wei, Junichi Inaba, Yan Zhao, Joseph D. Mowery, Rosemarie Hammond

**Affiliations:** 1 Molecular Plant Pathology Laboratory, Beltsville Agricultural Research Center, Agricultural Research Service, United States Department of Agriculture, Beltsville, MD 20705, USA; junichi.inaba@usda.gov (J.I.); yan.zhao@usda.gov (Y.Z.); rose.hammond@usda.gov (R.H.); 2 Electron and Confocal Microscopy Unit, Beltsville Agricultural Research Center, Agricultural Research Service, United States Department of Agriculture, Beltsville, MD 20705, USA; joseph.mowery@usda.gov

**Keywords:** phytoplasma, witches’-broom, axillary bud initiation, axillary bud outgrowth, starch breakdown, chloroplast degradation, premature leaf senescence

## Abstract

Witches’-broom (WB, excessive initiation, and outgrowth of axillary buds) is one of the remarkable symptoms in plants caused by phytoplasmas, minute wall-less intracellular bacteria. In healthy plants, axillary bud initiation and outgrowth are regulated by an intricate interplay of nutrients (such as sugars), hormones, and environmental factors. However, how these factors are involved in the induction of WB by phytoplasma is poorly understood. We postulated that the WB symptom is a manifestation of the pathologically induced redistribution of sugar and phytohormones. Employing potato purple top phytoplasma and its alternative host tomato (*Solanum lycopersicum*), sugar metabolism and transportation, and the spatiotemporal distribution of phytohormones were investigated. A transmission electron microscopy (TEM) analysis revealed that starch breakdown was inhibited, resulting in the degradation of damaged chloroplasts, and in turn, premature leaf senescence. In the infected source leaves, two marker genes encoding asparagine synthetase (*Sl-ASN*) and trehalose-6-phosphate synthase (*Sl-TPS*) that induce early leaf senescence were significantly up-regulated. However, the key gibberellin biosynthesis gene that encodes ent-kaurene synthase (*Sl-KS*) was suppressed. The assessment of sugar content in various infected tissues (mature leaves, stems, roots, and leaf axils) indicated that sucrose transportation through phloem was impeded, leading to sucrose reallocation into the leaf axils. Excessive callose deposition and the resulting reduction in sieve pore size revealed by aniline blue staining and TEM provided additional evidence to support impaired sugar transport. In addition, a spatiotemporal distribution study of cytokinin and auxin using reporter lines detected a cytokinin signal in leaf axils where the axillary buds initiated. However, the auxin responsive signal was rarely present in such leaf axils, but at the tips of the newly elongated buds. These results suggested that redistributed sucrose as well as cytokinin in leaf axils triggered the axillary bud initiation, and auxin played a role in the bud elongation. The expression profiles of genes encoding squamosa promoter-binding proteins (*Sl-SBP1*), and BRANCHED1 (*Sl-BRC1a* and *Sl-BRC1b*) that control axillary bud release, as determined by quantitative reverse transcription (qRT)-PCR, indicated their roles in WB induction. However, their interactions with sugars and cytokinins require further study. Our findings provide a comprehensive insight into the mechanisms by which phytoplasmas induce WB along with leaf chlorosis, little leaf, and stunted growth.

## 1. Introduction

Phytoplasmas are minute, and exclusively intracellular bacteria that infect numerous plant species, and cause serious economic losses [1]. A phytoplasma infection induces a series of remarkable symptoms in host plants, mainly characterized by flower abnormalities, including virescence (flower petals turning green), phyllody (leafy flowers), and cauliflower-like inflorescence (CLI) [2,3]. Many phytoplasma-affected plants also exhibit witches’-broom (WB, excessive shoot branching) [2]. WB is always accompanied by little leaf, leaf chlorosis, and stunted growth. Several phytoplasma virulence factors have been identified in aster yellows witches’-broom phytoplasma (AYWB) and onion yellows phytoplasma (OY). They are AYWB derived SAP05, SAP11, and SAP54, and OY originated TENGU and phyllogen [4,5,6,7,8].

AYWB-SAP54 and OY-phyllogen have been shown to be responsible for the flower deformation and they are highly homologous to each other in their amino acid sequences [6,8]. SAP54 interacts with plant 26S proteasomal shuttle proteins RADIATION SENSITIVE23 (RAD23), leading to the degradation of MADS-box transcription factors SEPALLATA3 and APETALA1, key regulators that control flower organ development [9]. A growing number of flower deformation inducers homologous to AYWB-SAP54 and OY-phyllogen have been identified, suggesting that they are very conserved in diverse phytoplasmas [10]. In addition to effector proteins that induce flower deformation, four effector proteins associated with the WB symptom (TENGU, SAP05, SAP11, SAP54) have also been reported [4,5,7,11]. They are genetically very divergent. TENGU is a small protein that is transportable between phloem cells and can even be detected in the shoot apex of phytoplasma-infected plants. TENGU down-regulates genes involved in the auxin responsive pathway; however, its direct target(s) in plants have not yet been identified, thus the mechanism by which TENGU induces WB remains unclear [7]. Transgenic plants overexpressing SAP11 show a “shoot branching” phenotype. SAP11 suppresses jasmonic acid (JA) synthesis by binding and destabilizing class II CIN (CINCINNATA)-TCP (TEOSINTE-BRANCHED, CYCLOIDEA, PROLIFERATION FACTOR 1, and 2), but how WB is induced by SAP11 needs to be further elucidated [5]. Recent studies have revealed that AYWB-SAP05 and SAP54 in paulownia witches’-broom phytoplasma (PaWB-SAP54) interact with plant 26S proteasome non-ATPase regulatory subunits RPN10 and RPN3, respectively; this results in the degradation of squamosa promoter-binding proteins (SPLs) [4,11]. Some SPLs (SPL9/15) are known to inhibit axillary bud outgrowth [12].

In higher plants, shoot branching consists of two developmental stages: (i) the initiation and establishment of axillary buds in leaf axils, and (ii) the outgrowth of axillary buds. This entire process of shoot branching is controlled by a complex interplay of hormonal, nutritional, and environmental cues [13]. In addition to the three main phytohormones auxin, cytokinins, and strigolactones (SLs), many genes responsible for transcription and signal transduction are involved in the regulation of these two developmental stages [13]. Auxin is synthesized in the shoot apex and acts as a repressor for branching and determines apical dominance. SLs, as a shoot branching suppressor, enhance the inhibition of auxin on branching, whereas cytokinins promote plant branching [14]. Recently, sugar (sucrose) has been identified as a key player in the modulation of hormonal networks and triggers the initiation and outgrowth of axillary buds [15,16,17,18]. For example, in potato, sucrose promotes the accumulation of cytokinins and induces axillary bud outgrowth and shoot branching [18].

Floral deformations (virescence, phyllody, and CLI) are believed to be unique symptoms induced by phytoplasmas, but shoot branching is not, as it is also observed in plants infected with viruses, viroids, and fungi [19,20,21,22]. It is worth noting that phytoplasma-induced shoot branching is more severe. Our previous study has shown that in tomato (cv. Moneymaker, MM) infected with potato purple top (PPT) phytoplasma, axillary buds started initiating and outgrowing from each leaf axil (45 days post inoculation, dpi), leading to an excessive shoot branching appearance (60 dpi), like a broom, hence the name ‘witches’-broom’ (WB) [2]. A new independent study [23] found that in a tomato mutant, the PPT phytoplasma infection did not cause WB or stunting symptoms. More interestingly, the phytoplasma infection not only prolonged the life span of the mutant plants, but also delayed the senescence of plants. Such results indicated that the WB symptom may be attributed to the pathogenic disruption of plant sugar metabolism and/or hormonal balances. However, few studies have been conducted on how sugar, hormones (auxin, SLs, and cytokinins), and genes in these signaling pathways trigger axillary bud initiation and outgrowth caused by phytoplasmas [2,4,11,24]. Therefore, this study aimed to better understand how a phytoplasma infection induces the WB symptom and to elucidate the roles of sugar, hormones, and related genes in this process.

To reach this goal, in the present study, sugar content was measured in different tissues of PPT phytoplasma-infected tomato plants. In addition, the spatiotemporal distribution patterns of auxin and cytokinins were also investigated by employing transgenic auxin and cytokinin reporter lines. The results indicated that sugar metabolism was severely impaired and sucrose transportation through phloem was impeded, which led to sucrose reallocation to new sink tissues (leaf axils and axillary buds). Reduced phloem translocation due to the reduction of sieve pores caused by excessive callose deposition also demonstrated impaired sugar transport. Transmission electron microscopy (TEM) revealed that the starch breakdown was inhibited, and the degradation of chloroplasts occurred in the infected source leaves. The levels of cytokinins elevated in the leaf axils, which favored the initiation of new lateral buds. Based on these results, a working model was proposed to elucidate how a phytoplasma infection impairs sugar metabolism and transportation in plants, leading to leaf chlorosis, little leaf, stunting, root system reduction, and WB symptoms.

## 2. Results

### 2.1. PPT Phytoplasma Infection Induced Witches’-Broom Symptoms and a Reduced Root System in Tomato Plants

**Figure 1:**
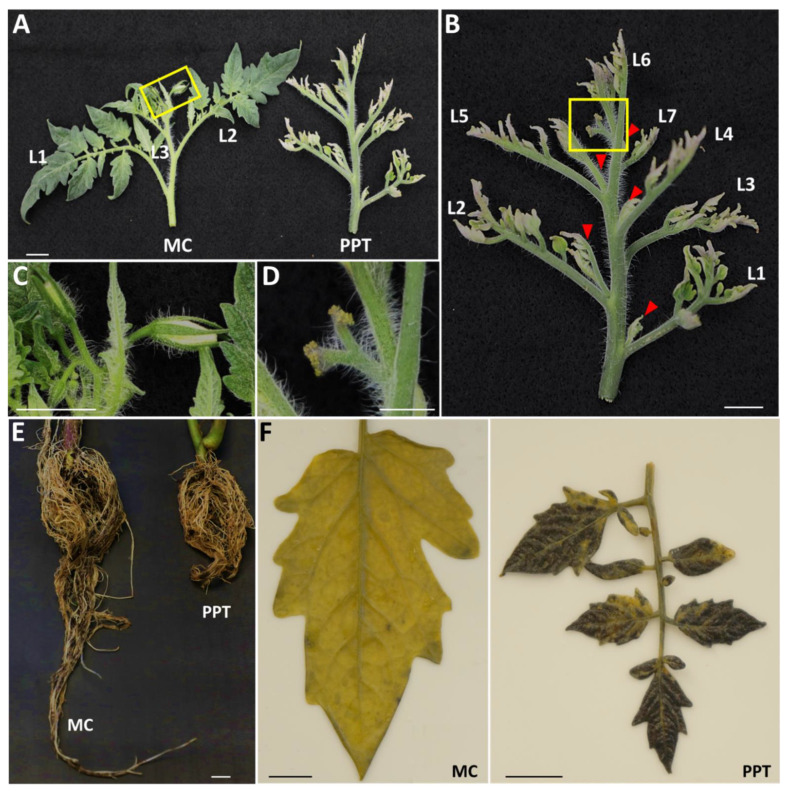
Potato purple top (PPT) phytoplasma infection-induced witches’-broom (WB), reduction in root system size and starch accumulation in leaf tissues. (**A**) PPT phytoplasma-induced typical WB in tomato plant (right panel) and a branch at the same developmental stage from mock control (MC) plant (left panel). (**B**) A closeup image of right panel of (**A**). Axillary buds initiated and outgrew from every leaf axil (shown by red triangles), exhibiting excessive branching. This was accompanied with little leaf and leaf chlorosis. Individual leaves are numbered. Yellow boxes indicate inflorescences and their closeup images are shown in (**C**,**D**). (**C**) MC inflorescence. (**D**) PPT phytoplasma-induced cauliflower-like inflorescence (CLI). (**E**) Root system reduction induced by PPT phytoplasma infection (shorter in length and loss of lateral roots, right panel) in contrast with roots of MC plant. (**F**) Lugol’s iodine solution staining for starch localization (purple pigments) in leaves of MC (left panel) and infected (right panel) plants. Scale bar = 1 cm.

**Figure 2:**
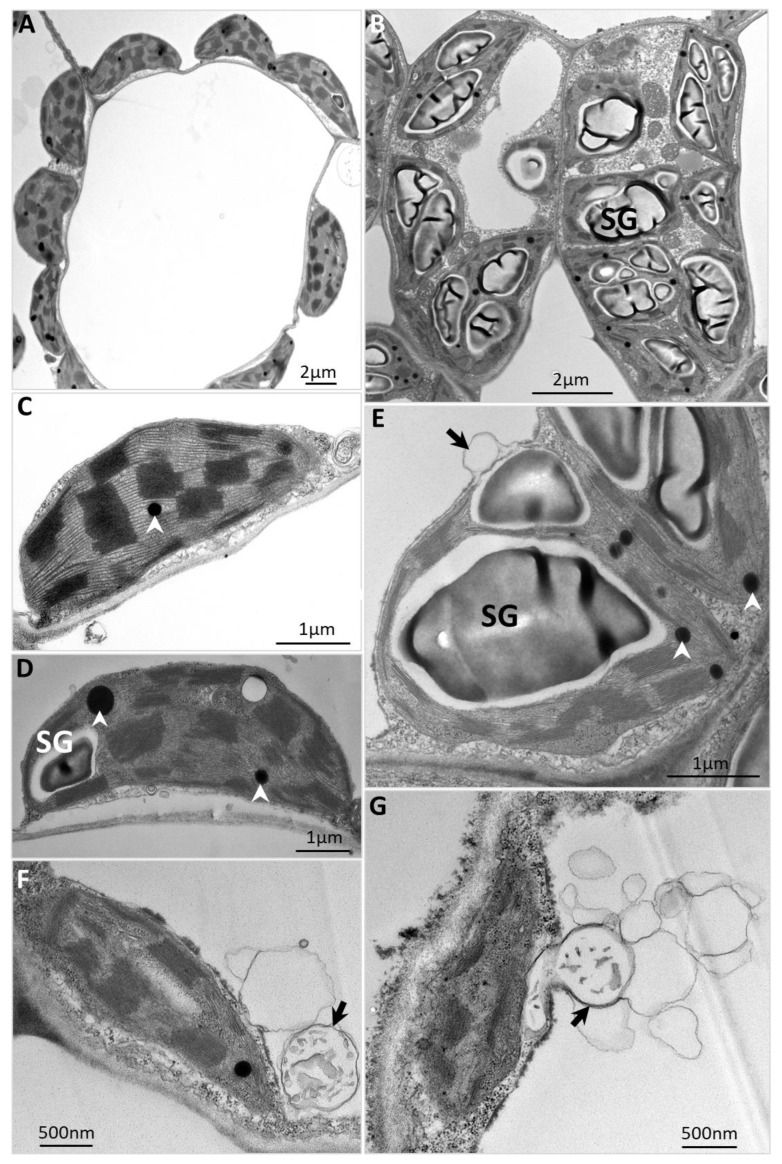
A morphological study of ultrastructure based on transmission electron microscopy revealed starch breakdown blockage and chloroplast degradation in plants induced by potato purple top (PPT) phytoplasma infection. (**A**) Well-organized multiple chloroplasts in the mesophyll cells of mock control (MC) plants. (**B**) Disorganized and misshapen multiple chloroplasts in the mesophyll cells of infected plants. (**C**,**D**) Chloroplasts in MC plants with tightly stacked thylakoids, containing either no (**C**) or only a flattened and discoid starch granule [SG, (**D**)]. (**E**) Deformed chloroplasts in the infected mesophyll cells. The swollen and irregular SGs occupied the entire chloroplast. (**F**,**G**) Damaged chloroplasts were degraded, and autophagosome-like structures (indicated by black arrows) were observed adjacent to the abnormal chloroplasts. White arrowheads indicate plastoglobules (lipids).

The PPT phytoplasma-infected tomato plants (cv. MM) exhibited a series of developmental symptoms such as big bud and cauliflower-like inflorescence (CLI) at about 28 and 45 dpi, respectively, and excessive initiation of axillary buds in the upper shoots appeared at 45 dpi (this study; [2]). In healthy tomato plants, axillary buds occurred only in the axils below the inflorescence for each sympodial unit. However, in infected plants, following the repeated initiation and outgrowth of axillary buds from every leaf axil, highly proliferative and compact branches formed at around 60 dpi, leading to the WB symptom (Figure 1A,B; [2]). Compared with the normal sympodial unit (three leaves and one inflorescence, Figure 1A,C), PPT phytoplasma-induced WB produced more leaves and CLI, (Figure 1D). Foliage in the WB structure was smaller in size, for example, Leaf 1 (L1) was around 10.2 cm in the mock control plant, which was much bigger than that (4.7 cm) in WB (Figure 1A). These small leaves were upright and often accompanied by chlorosis, sometimes with purple edges (Figure 1A,B). Besides the above-ground symptoms, the infected root system was also significantly reduced (Figure 1E; Supplementary Figure S1 and Table S1). Compared with the mock controls, the number, length, and width of lateral roots (secondary and tertiary roots) of infected plants were apparently decreased (Supplementary Figure S1 and Table S1). These results indicated that the phytoplasma infection inhibited the growth and development of the root system.

### 2.2. Starch Breakdown Blockage and Chloroplast Degradation in Phytoplasma-Affected Source Leaves

To investigate sugar metabolism changes in phytoplasma-infected plants, the contents of starch (complex sugar, a polymer of glucose), glucose (simple sugar), and sucrose were measured. First, starch accumulation and distribution in mature leaves of phytoplasma-infected plants were studied by using a starch indicator (Lugol’s iodine solution). Stained starch (bluish purple color) was visualized in almost the entire infected leaves. However, in the leaves of the mock inoculated plants at the same developmental stage, only sporadic spots were observed with light bluish-purple staining (Figure 1F).

Further, a transmission electron microscopy (TEM)-based morphological study was performed to observe the cell ultrastructure of infected leaves. Unlike the well-organized chloroplasts of the mock control leaves (Figure 2A), which possessed tightly stacked thylakoids, and contained no or only a flattened and discoid starch granule (Figure 2C,D), the chloroplasts in infected leaves were severely damaged and disorganized whether the leaves were collected during the day or at the end of the darkness period (Figure 2B). The irregular and swollen starch granules occupied almost the entire misshapen and swollen chloroplast (Figure 2E). Such abnormal starch granules suggest that the starch could not be degraded properly, and their excessive accumulation resulted in damage to the chloroplasts, which was demonstrated by destruction of the chloroplast membrane, disruption of the thylakoid membrane system (Figure 2E,F), and increased plastoglobules (lipids, black dots indicated by white arrowheads in Figure 2E).

The injured chloroplasts often produce detrimental reactive oxygen species (ROS) that trigger chloroplast degradation by autophagy [25,26]. The autophagosome-like structures were observed in mesophyll cells of plants infected with PPT phytoplasma (Figure 2E–G), and some misshapen chloroplasts were also found to fuse with autophagosome-like structures (Figure 2E–G). These results revealed that the phytoplasma infection induced starch breakdown blockage and chloroplast degradation, which thus led to the premature leaf senescence. Leaf senescence is the terminal stage of leaf development, and chloroplast collapse is the earliest sign of leaf senescence [27]. As a result, the chlorophyll pigments were destroyed [28], and the leaf color gradually changed from green to yellow.

### 2.3. Sugar Contents in Different Tissues of Tomato (cv. MM) Infected with PPT Phytoplasma

**Figure 3:**
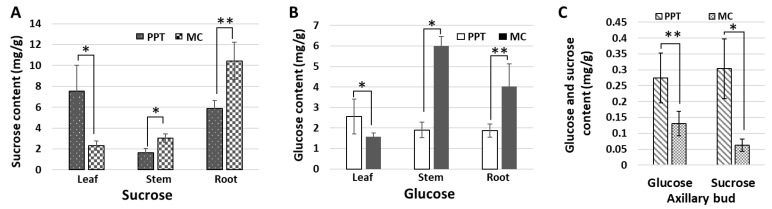
Sugar content measurement in different tissues of mock control (MC) and potato purple top (PPT) phytoplasma-infected tomato plants. (**A**,**B**) The contents of sucrose (**A**) and glucose (**B**) in leaf, stem, and root tissues of infected and MC plants. (**C**) Glucose and sucrose contents in newly released axillary buds (NR-AB) of infected and MC plants. Statistical significance * *p* < 0.01, ** *p* < 0.05.

In most plants, sucrose is the end-product of photosynthesis, and the primary sugar that is transported from the source tissues to the sink tissues through the phloem [29]. Plants rarely produce free glucose, as this monosaccharide is always converted to a stored form (starch) or combined with fructose to form sucrose [30]. In this study, the contents of glucose and sucrose in mature leaves, stems, roots, and newly released axillary buds (NR-ABs) were measured. The sucrose content was 7.53 ± 2.48 mg/g in the infected mature leaves, which was 3.25 times higher than that of the mock control leaves at the same developmental stage (2.31 ± 0.47 mg/g, Figure 3A). However, sucrose in the infected roots (5.85 ± 0.76 mg/g) was much lower than that of the mock control plants (10.44 ± 1.77 mg/g) (Figure 3A). In a stem, phloem is the channel through which sucrose is transported from the source tissues to the sink tissues. Thus, the main stem (around 0.5–1 cm below the leaf axil) was selected to determine the content of sucrose (transport sugar). The sucrose content was nearly two times lower in the stems of infected plants than in the mock controls (Figure 3A). In addition, a higher amount of sucrose was found in infected NR-ABs compared with the mock controls (Figure 3C). As with sucrose, glucose content was also high in source leaves and NR-ABs (Figure 3B,C), and low in stems and root tissues (Figure 3B). Sucrose content was high in source leaves and low in main stems and sink roots, indicating that the sucrose transportation through phloem was severely reduced or hampered in plants infected with phytoplasmas.

### 2.4. Phytoplasma Infection-Induced Excessive Callose Deposition in the Phloem

**Figure 4:**
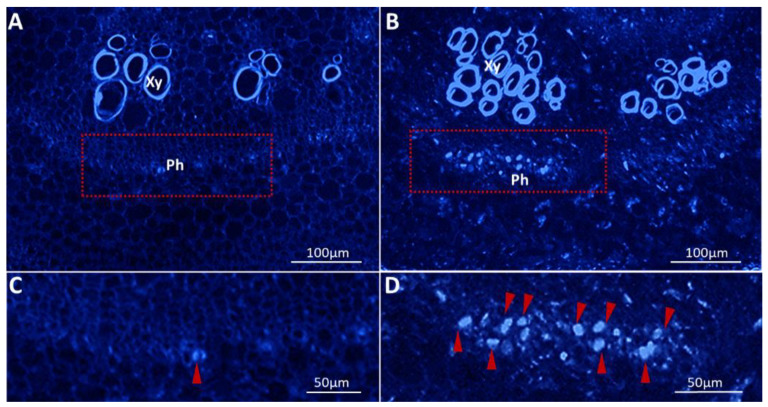
Aniline blue staining of callose deposition in the tomato plants induced by potato purple top (PPT) phytoplasma. (**A**,**B**) Cross stem sections of mock control and infected plants, respectively. (**C**,**D**) Closeup images of red rectangle boxes in (**A**,**B**), respectively. Red triangles show the callose deposition (bright blue fluorescence) in the phloem (indicated by Ph). Xy represents xylem.

Callose (β-1,3-glucan) is a linear plant polysaccharide. Callose deposition in the phloem (sieve plate pores) can reduce phloem conductivity and impact the intercellular connections [31]. Pathogen-induced callose deposition is irreversible and provides the physical barriers to cells, sealing off the injured plant tissue [31]. In this study, the accumulation of callose in the phloem of the phytoplasma-infected tomato plant was studied by confocal fluorescence microscopy of ultrathin paraffin sections (cross stem sections). High intensity of bright blue fluorescence (callose stained with aniline blue) was observed in the PPT phytoplasma-infected phloem (Figure 4B,D), while specific callose staining was rarely found in the mock controls (Figure 4A,C). Furthermore, the fluorescence intensity of callose was assessed based on the histogram function of Adobe Photoshop software. The average fluorescence intensity value of infected plants was 141.24 ± 9.28, which was 2.13-fold higher than that (66.19 ± 16.79) in control plants. According to the pixel values, the stained callose spots in infected plants were also 2.89 times bigger than controls (Supplementary Table S2).

**Figure 5:**
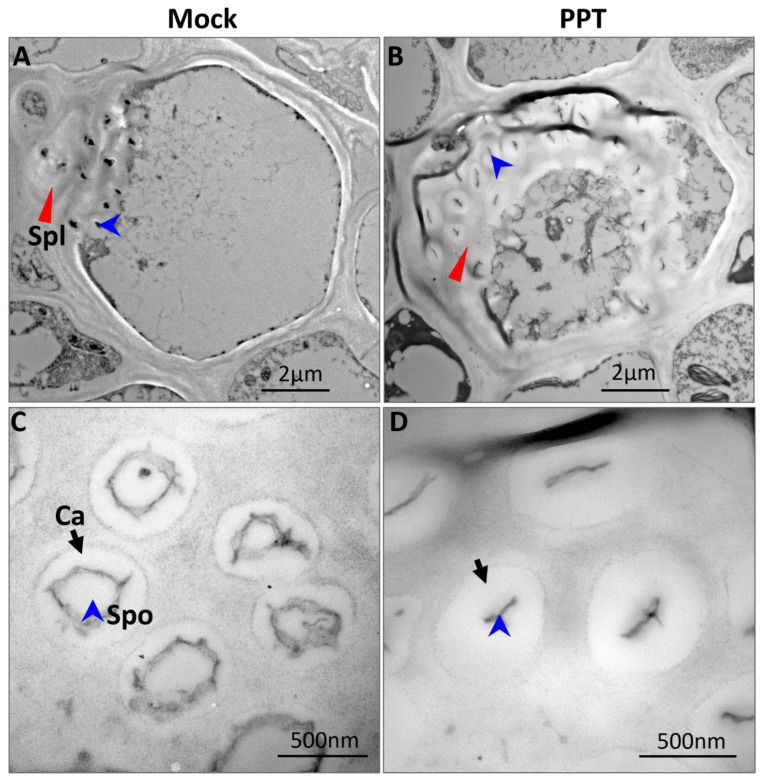
Transmission electron microscopy (TEM) analysis revealed excessive callose deposition in the sieve plates of tomato plants induced by potato purple top (PPT) phytoplasma. (**A**,**B**) Sieve elements with sieve plates in mock control (**A**) and PPT phytoplasma-infected (**B**) plants. The red triangles and blue arrowheads point to sieve plate (Spl) and sieve pore (Spo), respectively. (**C**,**D**) Sieve pores (indicated by blue arrowheads) in mock control (**C**) and infected (**D**) plants. Black arrows indicate callose (Ca) deposition.

In addition, a TEM analysis also revealed a larger amount of callose deposition around the sieve pores of infected plants (Figure 5B,D), which almost sealed the sieve pores, making the pores smaller and narrower (Figure 5D). In the mock control plants, although there was a small amount of callose deposition, it did not affect the size of sieve pores (Figure 5A,C). These results suggested that phytoplasma infection-induced excessive callose deposition resulted in the reduction in phloem translocation. This supports the observation that the transport of sucrose through phloem was severely hampered and may explain why the sucrose content is high in the source leaves and low in the sink tissues (roots) of phytoplasma-infected plants in contrast to mock control plants.

### 2.5. Spatiotemporal Distribution of Plant Hormones in PPT Phytoplasma-Infected Transgenic Reporter Tomato Plants

**Figure 6:**
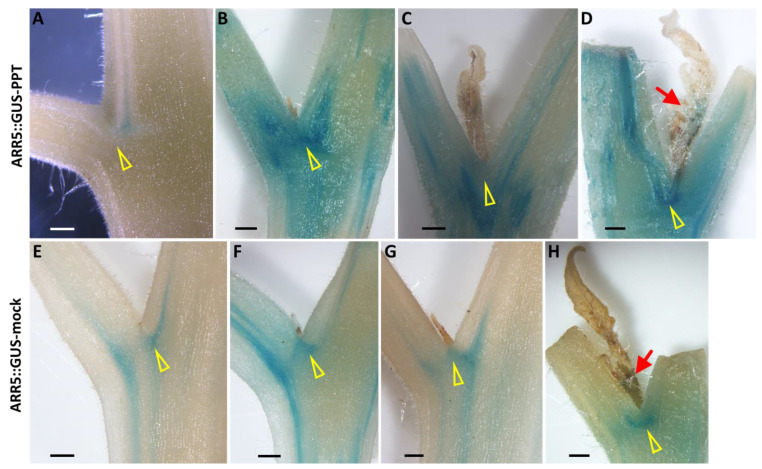
Spatial and temporal distribution patterns of cytokinin responsive signal in mock control and potato purple top (PPT) phytoplasma-infected ARR5::GUS transgenic tomato plants. (**A**–**D**) PPT phytoplasma-infected ARR5::GUS plants. (**A**) Lightly GUS-stained leaf axil (low cytokinin level, no axillary bud initiation was observed, LA-NO-AB). (**B**) Heavily GUS-stained leaf axil where a new axillary bud initiated, LA-AB. (**C**) A newly released and outgrown bud where cytokinin signal was rarely distributed in leaf axil. (**D**) A newly elongated branch with a new inflorescence (indicated by a red arrow). (**E**–**H**) Mock control ARR5::GUS plants. (**E**) Low intensity of GUS staining in mock LA-NO-AB. (**F**–**H**) Cytokinin distribution at the leaf axils in a newly initiated bud (**F**), a newly released and outgrown bud (**G**), and a newly elongated branch with a newly initiated inflorescence (indicated by a red arrow). Yellow triangles indicate the leaf axils. Scale bar = 0.5 mm.

**Figure 7:**
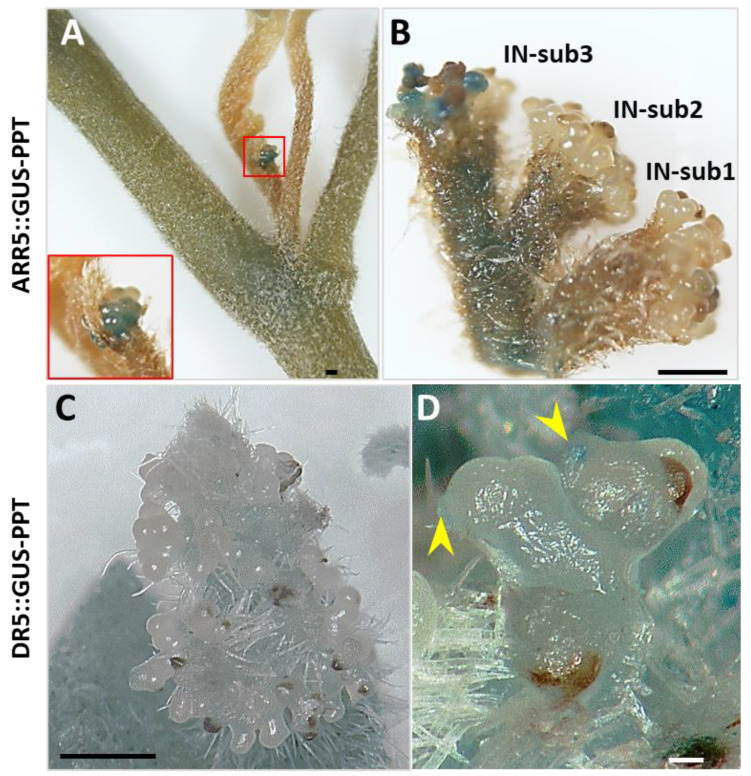
Spatial and temporal distribution patterns of cytokinin and auxin signals in potato purple top (PPT) phytoplasma-infected ARR5::GUS and DR5::GUS tomato transgenic plants. (**A**,**B**) ARR5::GUS cytokinin reporter line. (**A**) An outgrown branch with a new cauliflower-like inflorescence (CLI). Big red box is a closeup image of the small red box, showing indeterminate inflorescence meristems with a high level of cytokinin. (**B**) Cytokinin signal sequentially triggered the inflorescence branch development from inflorescence subbranch (IN-sub) 1 to 3. (**C**,**D**) DR5::GUS auxin reporter line. (**C**) PPT phytoplasma infection-induced CLI in DR5::GUS line. GUS staining (auxin signal) was not observed in CLI structure which is formed by indeterminate inflorescence meristems. (**D**) Occasionally, GUS staining (indicated by yellow arrowheads) could be visualized in floral meristems. Scale bar = 0.5 mm.

Arabidopsis response regulator 5 (ARR5), a response factor in the cytokinin signaling pathway, is induced by cytokinin. In many studies, the ARR5::GUS (β-glucuronidase) reporter construct has been used as a tool to localize active cytokinin [32]. In PPT phytoplasma-infected tomato plants, the cytokinin responsive signal was investigated at four different developmental stages during axillary bud initiation and outgrowth, including (i) leaf axil (bud not yet initiated), (ii) leaf axil (bud just initiated), (iii) newly initiated and outgrown bud, and (iv) newly elongated bud. Intense blue GUS staining was observed in the leaf axils where new axillary buds (LA-AB) could be formed (Figure 6B; Supplementary Figure S2), representing the ARR5 responsiveness to a large amount of cytokinin distribution, whereas only light blue GUS staining was visualized in the leaf axils where no axillary buds (LA-NO-AB) initiated (Figure 6A; Supplementary Figure S2). The intensity value (78.55 ± 1.25) in PPT-LA-AB was obviously stronger than that (143.90 ± 9.02) in PPT-LA-NO-AB (the GUS staining intensity was stronger, and the RGB value was lower because RGB white was RGB 255,255,255; RGB black was RGB 0,0,0. Supplementary Figure S2). In mock control plants, the intensity of GUS staining in LA-ABs (Figure 6F; Supplementary Figure S2) was slightly stronger than that in LA-NO-ABs (Figure 6E; Supplementary Figure S2), but the difference was not significant. In addition, it was obvious that a higher level of the cytokinin signal was detected in PPT-LA-ABs than mock-LA-ABs (Figure 6B,F; Supplementary Figure S2). Interestingly, cytokinin occurred at the leaf axils where the axillary buds initiated, but not in the axillary buds themselves (Figure 6C,G; Supplementary Figure S2). Only when a new branch was about to form an inflorescence did cytokinin signals move to the inflorescence and trigger the initiation and division of inflorescence meristems (Figure 6D,H and Figure 7A,B; Supplementary Figure S2).

**Figure 8:**
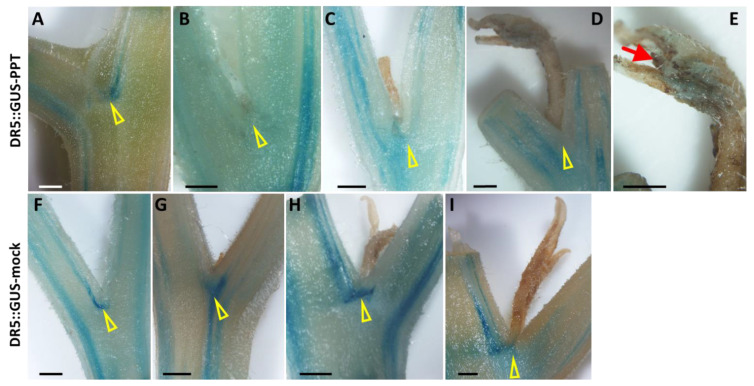
Spatial and temporal distribution patterns of auxin responsive signal in mock control and potato purple top (PPT) phytoplasma-infected tomato DR5::GUS transgenic lines. (**A**–**E**) PPT phytoplasma-infected DR5::GUS plants. (**A**) Lightly GUS-stained leaf axil (low auxin level, no axillary bud initiation was observed, LA-NO-AB). (**B**) No apparent GUS-stained leaf axil where a new axillary bud initiated, LA-AB. (**C**) A newly released and outgrown bud (no significant GUS signal was observed at the leaf axil). (**D**) An outgrown and elongated branch. No apparent GUS staining was found at the leaf axil, but auxin signal localized in the tip of the new branch. (**E**) Close up image of newly outgrown and elongated branch in (**D**), and red arrow shows the blue GUS staining. (**F**–**H**) Mock control DR5::GUS plants. (**F**) Auxin signal in mock-LA-NO-AB. (**G**,**H**) Auxin signal occurred in the infected leaf axils of a newly initiated bud (**G**), a newly released and outgrown bud (**H**), and a newly outgrown and elongated branch (**I**). Yellow triangles indicate the leaf axils. Scale bar = 0.5 mm.

DR5 is a highly active synthetic auxin response element. A DR5::GUS reporter system has been widely used for the visualization of localized auxin responses [33]. Employing the DR5::GUS reporter line, the auxin responsive signal was also examined at four different developmental stages: (i) leaf axil (bud not yet initiated); (ii) leaf axil (bud just initiated); (iii) newly initiated and outgrown bud; and (iv) newly elongated bud. Light blue staining which reflects the auxin distribution was observed in mock-LA-NO-ABs and PPT-LA-NO-ABs (Figure 8A,F; Supplementary Figure S3). Strong blue GUS staining was observed in the leaf axils of newly initiated and released axillary buds of mock control plants (Figure 8G,H; Supplementary Figure S3), while no significant signal responsive to auxin was present in such buds of phytoplasma-infected plants (Figure 8B,C; Supplementary Figure S3). Considering the role of auxin in inhibiting axillary bud release, these results suggest that from initiation to release and elongation phytoplasma infection-induced axillary buds do not undergo the dormancy that healthy (mock) plants normally do. In addition, auxin localization could be visualized in newly outgrown and elongated infected axillary buds, especially in bud tips (Figure 8D,E), while not in the mock buds which were at the same developmental stage (Figure 8I; Supplementary Figure S3).

**Figure 9:**
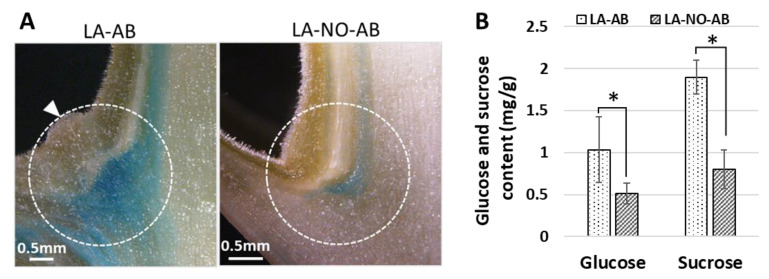
Distribution of cytokinin responsive signal and sugar measurement in leaf axils of ARR5::GUS cytokinin reporter tomato plants infected with potato purple top (PPT) phytoplasma. (**A**) Intense GUS staining (reflecting high level of cytokinin) in leaf axil where a new axillary bud initiated, LA-AB (leaf panel) and light GUS staining (a small amount of cytokinin signal) in leaf axil where no axillary bud initiated, LA-NO-AB (right panel). The newly initiated bud was indicated by a white triangle. Leaf Axils (LA-ABs and LA-NO-ABs) with white circles were sampled for sugar content assessment. (**B**) Glucose and sucrose contents in leaf axils (LA-ABs and LA-NO-ABs) of PPT phytoplasma-infected plants. Statistical significance * *p* < 0.01.

In this study, the ARR5::GUS tomato transgenic line was also employed to investigate the sugar content in leaf axils. Sucrose content in the LA-ABs (1.89 ± 0.12 mg/g) was much higher than that in the LA-NO-LBs (0.80 ± 0.23 mg/g) of PPT phytoplasma-infected plants (Figure 9B). These results strongly suggested that high amounts of sucrose accumulated in the leaf axils of infected plants, triggering the initiation of axillary buds. However, there was no significant difference in sugar content between LA-ABs and LA-NO-ABs in mock control plants (Supplementary Figure S4B). This was consistent with distribution patterns of cytokinin and auxin in the LA-ABs and LA-NO-ABs of mock controls (Figure 6E–H and Figure 8F–I; Supplementary Figures S2–S4).

### 2.6. Expression Profiles of Genes Involved in Premature Leaf Senescence, Gibberellin Synthesis, and Axillary Bud Release

**Figure 10:**
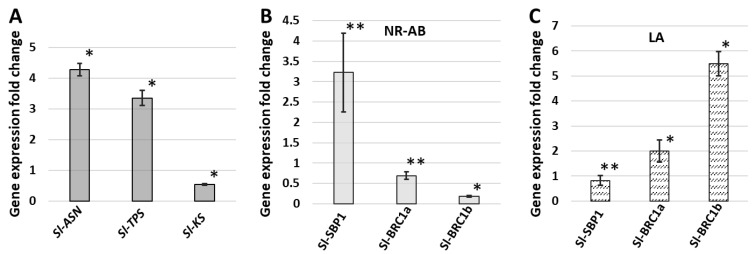
Expression profiles of marker genes involved in early leaf senescence, gibberellin synthesis, and axillary bud outgrowth of tomato plants infected with potato purple top (PPT) phytoplasma. (**A**) Expressions of marker genes of early leaf senescence, genes that encode asparagine synthetase (*Sl-ASN*) and trehalose-6-phosphate synthase (*Sl-TPS*), and a gene that encodes ent-kaurene synthase (*Sl-KS*), a key enzyme for gibberellin synthesis. (**B**,**C**) Expressions of genes that control axillary bud outgrowth in (**B**) newly released axillary buds (NR-AB) and (**C**) leaf axil (LA). Genes include *Sl-SBP1* (tomato ortholog of arabidopsis squamosa promoter-binding proteins (SPLs)) and *BRC1a* and *BRC1b* (tomato orthologs of arabidopsis BRANCHED-1). Gene expression fold changes in (**A**,**B**) determined by real time PCR were relative to those of the mock-inoculated controls (a value of 1.00 was assigned). Gene expression fold changes in (**C**) were the relative changes between leaf axils where axillary buds initiate (LA-AB) and the leaf axils where no axillary buds initiate (LA-NO-AB, a value of 1.00 was assigned). The β-Actin gene was used as an internal reference. Up- or down-regulations of all differentially expressed genes are statistically significant (* *p* < 0.01, ** *p* < 0.05).

A TEM-based morphological analysis indicated PPT phytoplasma infection induced starch breakdown blockage and premature leaf senescence. In Arabidopsis, two genes that encode asparagine synthetase (ASN) and trehalose-6-phosphate synthase (TPS) are induced during early leaf senescence [34,35]. In addition, it has been reported that inefficient starch metabolism severely reduced the expression of a gene that encodes ent-kaurene synthase (KS), a key enzyme for gibberellin synthesis [36]. In the present study, the expressions of tomato orthologs (*Sl-ASN*, *Sl-TPS*, and *Sl-KS*) were investigated in infected plants by quantitative reverse transcription (qRT)-PCR. Both *Sl-ASN* and *Sl-TPS* were significantly up-regulated in infected mature leaves (Figure 10A). This result confirmed PPT phytoplasma induced early senescence, which was consistent with the results of the TEM analysis. In addition, the *Sl-KS* gene was down-regulated in infected plants (Figure 10A), suggesting a reduction in gibberellin synthesis.

In Arabidopsis, BRANCHED1 (BRC1, SLs marker gene) and squamosa promoter-binding proteins (SPLs) have been reported to control axillary bud outgrowth [12,37]. In this study, the expression patterns of three tomato orthologs *Sl-BRC1a*, *Sl-BRC1b*, and *Sl-SBP1* (SPL9/15 in Arabidopsis) in the LA-ABs and newly released axillary buds (NR-ABs) were determined by qRT-PCR. *Sl-BRC1a* and *Sl-BRC1b* were up-regulated, and *Sl-SBP1* was slightly down-regulated in LA-ABs compared with LA-NO-ABs (Figure 10C). On the other hand, *Sl-BRC1a* and *Sl-BRC1b* were down-regulated, and *Sl-SBP1* was up-regulated in infected NR-ABs compared with NR-ABs in the mock control plants (Figure 10B).

## 3. Discussion

Shoot branching refers to the formation/activation of axillary buds in leaf axils and development of new flowers or branches. It is an important agronomic trait, which strongly affects the cultivation suitability, yield, and harvest efficiency of plants, especially agricultural and horticultural crops [38]. The entire process of shoot branching is complex, involving a series of fine tuning of endogenous (nutritional and hormonal) and exogenous (environmental) signals. Among these signals, auxin and SLs behave as repressors, while sugar and cytokinins are considered to be inducers [14]. Sugar has been recognized in recent years as an early modulator that integrates downstream hormone signaling pathways and activates axillary bud release [15,16,18]. However, it is unknown if and how sugar triggers the phytoplasma-induced excessive release of axillary buds from each leaf axil. In phytoplasma-infected plants, the accumulation of various carbohydrates in diseased leaves and the alterations in the sugar metabolism pathway have been reported [39,40,41]. However, there is still a lack of a comprehensive understanding of sugar metabolism and sucrose transportation in phytoplasma-infected plants.

In this study, irregular and swollen starch granules were observed in the misshapen chloroplasts of PPT phytoplasma-infected leaves (Figure 2E). The starch granules occupied almost the entire chloroplast, presumably because the starch was not metabolized, resulting in damaged and dysfunctional chloroplasts. The subsequent consequence was the severe loss of chlorophyll [26]. Due to a lack of chlorophyll, infected leaves showed apparent chlorosis (leaf yellowing, Figure 1A,B). Such a leaf yellowing symptom was also exhibited in many other plants affected by phytoplasmas [42,43].

The starch breakdown (decomposition) in infected source leaves was blocked, leading to the degradation of damaged chloroplasts (Figure 2F,G), which is a sign of premature leaf senescence. Premature leaf senescence was also demonstrated by the up-regulation of the early leaf senescence marker genes *Sl-ASN* and *Sl-TPS* in PPT phytoplasma-infected mature leaves (Figure 10A). Furthermore, the blocked starch breakdown also affected the synthesis of the growth-promoting phytohormone gibberellin, which controls plant height [44]. In infected leaves, the gene that encodes ent-kaurene synthase (KS), a key enzyme in gibberellin synthesis, was significantly down-regulated (Figure 10A). This result was consistent with the findings of our previous study, that is, the content of gibberellin (GA) in a PPT phytoplasma-infected tomato plant was lower than that of the mock controls, and GA metabolism genes *GA20ox1* and *GA3ox1* were significantly down-regulated [45]. Impaired GA synthesis and metabolism are thought to be the cause of stunted growth. In addition, many early senescence mutants exhibited a retarded growth phenotype, with reduced plant height (dwarfism or semi-dwarfism), withered leaf tip, and decreased chlorophyll content [46]. In summary, leaf chlorosis, little leaf, and stunted growth are a series of consequences of premature leaf senescence caused by phytoplasma-induced sugar metabolism disorders.

In addition to sugar metabolism, sugar transport in phytoplasma-infected plants was also studied. Sugar moves from source to sink by long-distance translocation in the phloem. Mature leaves are the source and active growing parts such as shoot apex, roots, and reproductive organs are the sink [47]. Shoot branching has been considered to be related to source–sink status [17]. In PPT phytoplasma-infected plants, sucrose and glucose levels in mature leaves (source tissue) were higher and decreased in stems (the intermediate channel between source and sink tissues) and roots (sink tissue) (Figure 3). These results suggested that sucrose transportation through the phloem was severely slowed down or blocked. This is well demonstrated by the excessive deposition of callose in the phloem and reduction in sieve pore size induced by PPT phytoplasma (Figure 4B,D and Figure 5B,D). Moreover, along with low sucrose and glucose contents, the growth and development of the infected roots significantly reduced as well (Figure 1E; Supplementary Figure S1 and Table S1). Sucrose and glucose have been known as signaling molecules for promoting root growth and development, including root elongation and lateral root formation [48,49]. Therefore, the low sugar content in roots is likely to be responsible for the PPT phytoplasma-induced root system reduction in size.

In plants, sugar (sucrose) is redistributed and accumulates in the leaf axils. When the concentration surpasses a certain threshold, the axillary bud release will be activated. This has been demonstrated in peas by a radiolabeling technique and exogenous sugar application [15]. Recently, sucrose has been reported to promote stem branching through cytokinin [18]. Such findings prompted us to investigate whether sugar and cytokinin play roles in the phytoplasma-induced release of axillary buds. In the present study, sugar content and cytokinin distribution in the leaf axils were examined. Employing the ARR5::GUS (cytokinin) reporter tomato line, the leaf axil infected with PPT phytoplasma was cut in half. One half was used for sugar measurement, and the other half was used for GUS staining. A strong GUS signal (a large amount of cytokinin) was observed in the leaf axil where the axillary bud (LA-AB) would initiate, and a weak GUS signal (small amount of cytokinin) was visualized in the leaf axil where no axillary bud (LA-NO-AB) initiated (Figure 9A).

Consistent with cytokinin distribution (Figure 9A), the sucrose content in LA-AB was significantly higher than that in the LA-NO-AB of PPT phytoplasma-infected plants (Figure 9B). Such a result strongly indicated that sucrose was remobilized, and accumulated in leaf axils, triggering axillary bud initiation induced by phytoplasma. Combining all the results of sugar content measurement in the leaf axils (Figure 9B) and various plant tissues (Figure 3), we concluded that phytoplasma infection disrupted the long-distance transport of sucrose through phloem, which led to sucrose reallocation and an altered sucrose transport pathway, i.e., the final destination of transport was changed from roots to leaf axils and axillary buds.

It is worth noting that there were no significant variances in the sugar content or cytokinin distribution between the LA-ABs and LA-NO-ABs of mock control plants (Supplementary Figures S2 and S4). Furthermore, in the DR5::GUS (auxin) reporter tomato line, different auxin distribution patterns in the mock and infected leaf axils were identified (Figure 8; Supplementary Figure S3). Auxin can inhibit the release of axillary buds localized in mock-LA-ABs (Figure 8G,H), but very little was visualized in PPT-LA-ABs (Figure 8B,C). This was consistent with the observation that PPT phytoplasma infection induces the initiation of axillary buds and promotes their release and elongation, while newly initiated healthy (mock) axillary buds tend to remain dormant. Further girdling or radiolabeling experiments or the exogenous sugar supply will elucidate the roles of sugar and cytokinin in initiating and activating axillary buds in healthy tomato plants.

Although the cytokinin signal was visualized in the LA-ABs of both mock control and infected plants (Figure 6B,F), it was not in the newly released buds (Figure 6C,G). Auxin was rarely identified in the infected LA-ABs (Figure 8B,C), but interestingly, auxin signal was found in the infected newly elongated buds (Figure 8D,E), particularly in the bud tips (Figure 8E). In PPT phytoplasma-induced CLI structures, cytokinin distribution was also found in the differentiating and dividing inflorescence meristems (Figure 7A,B), but auxin was undetectable except occasionally in the floral meristem (Figure 7C,D). These results indicate that cytokinins play a pivotal role in the initiation and division of various developmental processes such as the activation of axillary bud initiation, inflorescence meristem differentiation and division, as well as inflorescence branching. Auxins promote elongation, including the elongation of axillary buds and further development of floral meristems. The distribution pattern of cytokinins and auxins in abnormal flower buds (big buds) induced by PPT phytoplasma also supports the above conclusion. For example, strong cytokinin signaling was localized in undifferentiated carpel primordia at stage 6 of flower bud development induced by PPT phytoplasma. Once the individual stamen and carpel primordium were established and positioned (stage 8), strong auxin signals were observed (unpublished data), suggesting the role of auxin in promoting the further development of reproductive flower organs. Moreover, excessive axillary bud initiation and CLI induced by phytoplasma infection implicated the possibility that phytoplasma can be used as an effective tool to study plant morphogenesis.

It is noteworthy that DR5 and ARR5 are the response elements of auxin and cytokinin, respectively. Their activities are triggered by the corresponding hormones, which are either synthesized in situ or incoming-transported. This suggests that hormone synthesis and/or transport may play roles in the development of a WB symptom induced by phytoplasma infection. Future studies on biosynthesis, metabolism, and transport of phytohormones will help us to understand their roles in the development of phytoplasma-induced symptoms.

Several marker genes involved in axillary bud initiation and outgrowth were also investigated in tomato plants infected with PPT phytoplasma. *Sl-BRC1a* and *Sl-BRC1b*, (key repressors of branching, negatively regulated by SLs), showed very interesting spatiotemporal expression pattens. Both *Sl-BRC1a* and *Sl-BRC1b* were up-regulated in the LA-ABs compared with the LA-NO-ABs of infected plants (Figure 10C). Cytokinins are known to promote BRC1 transcription in activating axillary buds in peas [35]. In LA-ABs, the cytokinin-induced strong intensity of the ARR5::GUS signal was consistent with the up-regulated transcriptions of BRC1a and BRC1b. It is well known that the *BRC1* gene is mainly expressed in axillary buds and inhibits axillary bud outgrowth [50,51]. In other words, low transcription levels of BRC1 activate axillary bud outgrowth. Compared with the NR-ABs of mock control plants, *Sl-BRC1a* and *Sl-BRC1b* were down-regulated in infected NR-ABs (Figure 10B). This result strongly indicated that PPT phytoplasma-induced axillary buds were also released by inhibiting BRC1a and BRC1b transcription levels, similar to those of normal plants.

Two recent studies have reported that phytoplasma effectors PaWB-SAP54 and AYWB-05 interact with RPN10 and RPN3 (plant 26S proteasome subunits) and degrade SPLs proteins, resulting in the WB symptom [4,11]. In this study, the tomato SPL ortholog, *Sl-SBP1* was slightly down-regulated in the LA-ABs compared with the LA-NO-ABs (Figure 10C), while in infected NR-ABs, it was up-regulated (Figure 10B). In Arabidopsis, *SPL9* and *SPL15* directly interact with *FHY3* and *FAR1* (proteins involved in phytochrome A-mediated light signaling pathways) and *SMXL6*/*SMXL7*/*SMXL8* (SL repressors) to suppress *BRC1* [52]. Many SPL transcription factors are targeted by miR156, and sugar has been identified as an upstream component of the miR156/SPL module [53]. In the process of axillary bud release induced by PPT phytoplasma, high sucrose content in the LA-ABs was always accompanied by an increased cytokinin (Figure 9). Salam et al. [18] reported that sucrose promoted branching by activating cytokinin accumulation. In the initiation and outgrowth of axillary buds induced by PPT phytoplasma infection, whether *Sl-SBP1* (SPL) acts as a repressor of *SlBRC1a* and *SlBRC1b* and how sugar regulates these downstream genes to control bud outgrowth remains to be investigated.

In the WB structure, the axillary buds initiate from each leaf axil and then outgrow, but it is very apparent that these new buds cannot grow very large and stop expanding at a certain stage, forming compact small twigs like a broom (Figure 1A,B; [2]). Results from our previous and the present study indicated that the WB structure was formed by not only the repeated initiation and outgrowth of axillary buds but also the premature senescence. At the final phytoplasma infection stage, new meristems could barely differentiate, resulting in “drying out and dying” shoots at the top (data not shown). This must involve a series of synergistic effects, such as sugar and cytokinin supply, local auxin depletion, and the intricate regulation of many gene networks. Further study is needed to elucidate this mechanism.

**Figure 11:**
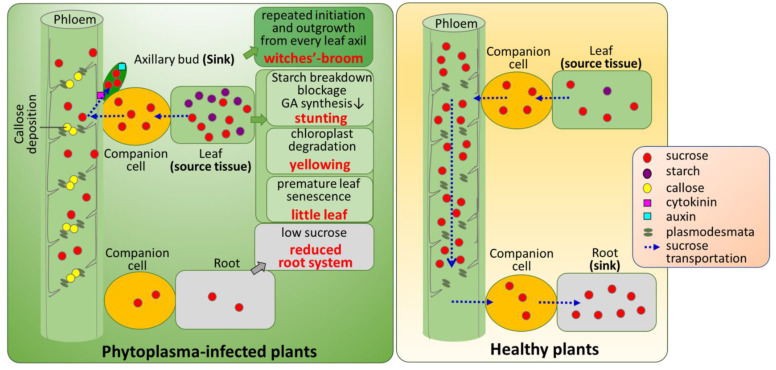
A proposed working model for symptoms induced by potato purple top (PPT) phytoplasma in the late infection stage. In this model, PPT phytoplasma infection induces starch breakdown blockage, and in turn degradation of damaged chloroplasts. This leads to premature leaf senescence, and the direct consequences are little leaf and leaf chlorosis. The blockage of starch breakdown also represses gibberellin (GA) synthesis, thus inhibiting plant growth and height. The PPT phytoplasma infection induces the reduction in sieve pore size due to the excessive callose deposition, which impedes sucrose translocation through phloem, resulting in the reallocation of sucrose in leaf axils. The high sucrose content in leaf axils triggers the initiation of the axillary bud. Cytokinin distribution in the leaf axils also participates in this bud initiation process. Auxin promotes elongation of the axillary buds. WB: witches’-broom; GA: gibberellin.

Based on the findings of this study, a working model was proposed to explain how phytoplasma infection induces changes in sugar metabolism and transportation and spatiotemporal distribution of phytohormones (Figure 11). Phytoplasma infection induces the blockage of starch breakdown and the degradation of dysfunctional chloroplasts. This results in premature leaf senescence and reduced GA synthesis, thus leaf chlorosis, little leaf, and stunted growth. Phytoplasma infection disrupts sugar metabolism and sucrose transportation, leading to the reallocation of sucrose to the leaf axil, where large amounts of cytokinin are distributed, activating the initiation of axillary buds. Repeated initiation and outgrowth of axillary buds and premature senescence lead to WB symptom.

## 4. Materials and Methods

### 4.1. Phytoplasma Strain, Plant Materials, and Graft Inoculation

The Columbia Basin PPT phytoplasma, also known as BLTVA, was identified in diseased potatoes from potato production fields in Washington and Oregon [54,55]. Tomato (cv. Moneymaker), transgenic DR5::GUS (auxin), and ARR5::GUS (cytokinin) reporter tomato lines (gifts from Dr. Eliezer Lifschitz, Technion-Israel Institute of Technology, Haifa, Israel) were used in this study. A PPT phytoplasma infection in tomato plants was established by grafting inoculation as previously described [2,3]. The experimental plants were maintained in a greenhouse with 16 h of light and 8 h of darkness. The light intensity ranged from 230 to 575 µmol/S/m^2^, corresponding to the seasonal variation of natural daylight throughout the year, which provided the best condition for growth and development of tomatoes in the greenhouse.

### 4.2. Lugol’s Iodine Staining

At 60 dpi, the mature leaves of tomato plants (cv. MM) infected with PPT phytoplasma and mock inoculated controls were collected during the day and at the end of darkness period. The collected leaves were cleared in 70% ethanol at 80 °C for 15 min, and stained with Lugol’s solution (Sigma-Aldrich, St. Louis, MO, USA) for 10 min at room temperature, and then rinsed with water for 15 min. The stained leaves were observed and imaged with an Olympus SZX12 stereo zoom microscope (Olympus, Tokyo, Japan).

### 4.3. GUS Staining

GUS staining was carried out according to the protocol previously described with minor modifications [56]. Leaf axils and inflorescences from infected transgenic DR5::GUS reporter and ARR5::GUS tomato lines and mock control plants were collected, respectively. The samples were incubated in a GUS staining buffer (50 mM phosphate buffered saline [PBS], pH 7.2, 0.05% Triton X-100, 2 mM potassium ferrocyanide, 2 mM X-Gluc) for 4 h at 37 °C in the dark. The stained tissues were then bleached with a graded ethanol series at room temperature until chlorophyll was completely removed. The samples were then photographed with an Olympus SZX12 stereo zoom microscope (Olympus, Tokyo, Japan).

### 4.4. Sugar Extraction and Measurement in Various Tissues of Tomato Plants

At 60 dpi, the mature leaf, main stem, and root (50 mg each sample), and newly released axillary bud (10 mg) samples were collected from tomato plants infected with PPT phytoplasma and mock controls, respectively. In addition, the leaf axils from the transgenic ARR5::GUS tomato line infected with PPT phytoplasma and mock inoculated controls were collected and cut in half. One half was used for GUS staining and the other half for sugar measurement. Heavily GUS-stained leaf axils (axillary buds to be formed, 25 mg) and lightly GUS-stained leaf axils (axillary buds not to be formed, 25 mg) were pooled, respectively. Sugar was extracted from different plant tissue samples according to the procedure described by Dallagnol et al. 2012 [57]. The tissue samples were ground in liquid nitrogen, homogenized, and incubated with 80% aqueous ethanol at 70 °C for 90 min. After centrifugation at 15,000 g for 10 min, the supernatant was transferred to a new tube. The pellet was suspended again in 80% aqueous ethanol for re-extraction under the same conditions, and the obtained supernatant was combined with the previous supernatant and stored at −20 °C until sugar measurement. The sugar measurement was carried out using a sucrose assay kit (Abcam, Cambridge, UK), following the manufacturer’s protocol. The sucrose was converted to glucose by invertase, and optical density (colorimetric absorbance) at 570 nm was measured by an ND-8000 nanodrop (Thermo Fisher Scientific, Waltham, MA, USA). The standard curve of sucrose was generated from the series of diluted sucrose (0, 1, 2, 3, 4, 5, 6 nmol) and the obtained OD values. Similarly, a glucose standard curve was also created. Data were analyzed statistically with a two-sample *t*-test program (https://www.evanmiller.org/ab-testing/t-test.html (accessed on 26 August 2021)).

### 4.5. Aniline Blue Staining of Paraffin Sections

The main stems of about 0.5 cm were excised from tomato plants infected with PPT phytoplasma and mock inoculated controls. The stem samples were fixed with 4% paraformaldehyde, and then embedded in paraffin, and sectioned to 10 μm thicknesses with a microtome (Leitz-1512 Microtome, Leitz, Wetzlar, Germany). Aniline blue staining was performed according to the procedure described by Ekawa and Aoki, 2017 [58]. Deparaffined cross stem sections were stained in 1% aniline blue solution (Thermo Fisher Scientific, Waltham, MA, USA) at room temperature for 30 min and rinsed with 1X PBS buffer three times. A Zeiss LSM710 confocal laser scanning microscopy (Carl Zeiss AG, Jena, Germany) system was utilized to capture fluorescent images utilizing an excitation wavelength of 405 nm with a broad filter set to detect all emission wavelengths between 410 and 513 nm. Images were captured using a Zeiss Axio Observer inverted microscope with a 10 × 0.5 NA Plan-Fluorite objective (Carl Zeiss AG, Jena, Germany). Zeiss Zen 2012 Pro software (Carl Zeiss AG, Jena, Germany) was used to obtain 40–150 z-stack images and maximum intensity projections. Based on the histogram function of Adobe Photoshop software, the fluorescence intensity of callose stained by aniline blue was assessed based on the histogram function of Adobe Photoshop software (https://helpx.adobe.com/photoshop/using/viewing-histograms-pixel-values.html (accessed on 26 August 2021)).

### 4.6. RNA Extraction and qRT-PCR Analysis of Gene Expression

Samples of mature leaves (50 mg), leaf axils (25 mg), and newly released buds (10 mg) were collected from the PPT phytoplasma-infected and mock-inoculated tomato plants at 60 dpi. Total RNAs were extracted by a hybrid protocol using the Trizol (Invitrogen, San Diego, CA, USA) and RNeasy (Qiagen, Valencia, CA, USA), as described previously [59]. First-strand cDNA was synthesized from 1 μg of the above extracted total RNA using an AffinityScript Multi Temperature cDNA synthesis kit, per the manufacturer’s instructions (Agilent Technologies, Santa Clara, CA, USA). qRT-PCR was carried out using Brilliant SYBR Green QPCR Master Mix (Agilent Technologies) and run on the STRATAGENE Mx3000P QPCR system (Stratagene, La Jolla, CA, USA). Primers were designed by online software Primer3 according to the sequences of tomato orthologs of genes that encode asparagine synthetase (ASN), trehalose-6-phosphate synthase (TPS), ent-kaurene synthase (KS), squamosa promoter-binding proteins (SPLs), and BRANCHED-1 (Supplementary Table S3). The primers for the internal control β-actin gene were designed previously [2]. Each qRT-PCR was performed with the thermocycling conditions as previously described [2]. Quantitation of the target gene expression was performed per the comparative cycle threshold (Ct) method (∆∆Ct method) [60]. Gene expression fold changes were relative to mock-inoculated control samples with the β-actin gene as an internal reference. For each sample type, at least three independent RNA samples from three plants (biological replicates) were subjected to the qRT-PCR analysis. Each experiment was repeated three times (technical replicates). Data were analyzed statistically using Student’s t test imbedded in Microsoft Excel (Microsoft Corporation, Seattle, WA, USA).

### 4.7. Transmission Electron Microscopy

Mature leaves (during the day and at the end of darkness period) and stems collected from PPT phytoplasma-infected tomato plants and mock control plants were cut into 1.5 mm pieces with a biopsy punch and fixed for two hours at room temperature in 2.5% glutaraldehyde, 0.05 M Na Cacodylate, 0.005 M CaCl_2_ (pH 7.0). The tissue was then rinsed five times with 0.05 M Na Cacodylate, 0.005 M CaCl_2_ buffer, and post-fixed in 1% buffered osmium tetroxide for two hours at room temperature. Afterwards, post-fixation samples were rinsed five times in the same buffer, dehydrated in a graded ethanol series followed by two exchanges of propylene oxide, infiltrated in a graded series of LX-112 resin/propylene oxide, and polymerized at 65 °C for 48 h. Silver-gold sections, with a thickness of 60–90 nm, were cut on a Reichert/AO Ultracut ultramicrotome with a Diatome diamond knife and mounted onto 100 mesh formvar/carbon-coated copper grids (Electron Microscopy Sciences, Hatfield, PA, USA). Grids were stained with 4% uranyl acetate and 3% lead citrate and imaged at 80 kV with a Hitachi HT-7700 transmission electron microscope (Hitachi, Tokyo, Japan).

## Data Availability

Not applicable.

## Supplementary Materials

The following are available online at https://www.mdpi.com/article/10.3390/ijms23031810/s1.
