# Supplementary material for: Phytoplasma Infection Blocks Starch Breakdown and Triggers Chloroplast Degradation, Leading to Premature Leaf Senescence, Sucrose Reallocation, and Spatiotemporal Redistribution of Phytohormones
Source: Int J Mol Sci. 2022 Feb 5;23(3):1810. doi: 10.3390/ijms23031810 (PMC8836287; doi:10.3390/ijms23031810)
Supplement: Supplementary file 1 [file ijms-23-01810-s001.zip › ijms-1569747-SI.pdf]

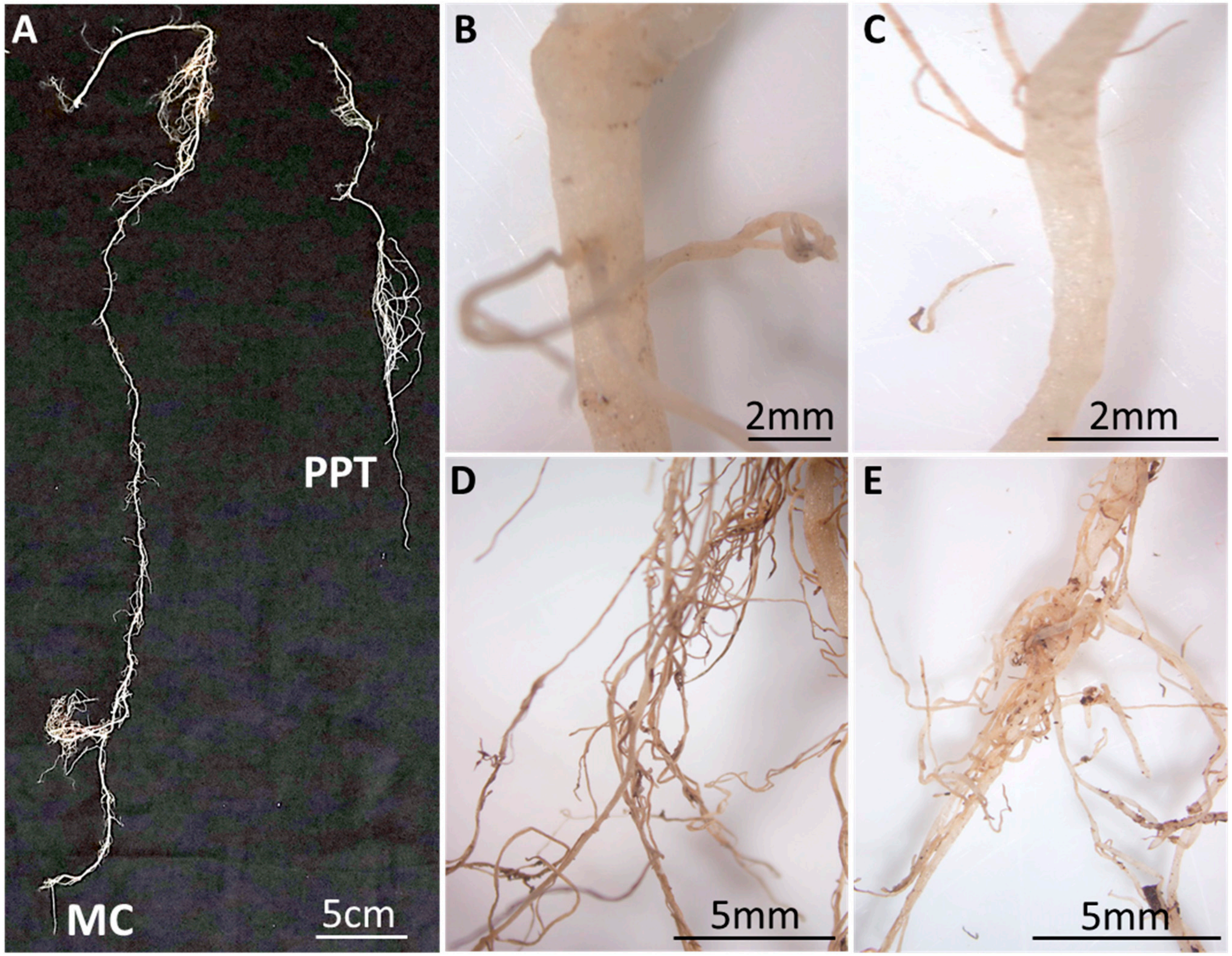


***Supplementary Figure S1.*** Comparison of the secondary and tertiary roots between mock control (MC) and potato purple top (PPT) phytoplasma-infected tomato plants. (A) A secondary root in MC (left panel) and PPT phytoplasma-infected (right panel) plants. (B-C) Comparison of the thickness of the base of the secondary root attached to the primary root between MC (B) and PPT-infected (C) plants. (D-E) Tertiary roots in MC (D) and PPT-infected (E) plants.

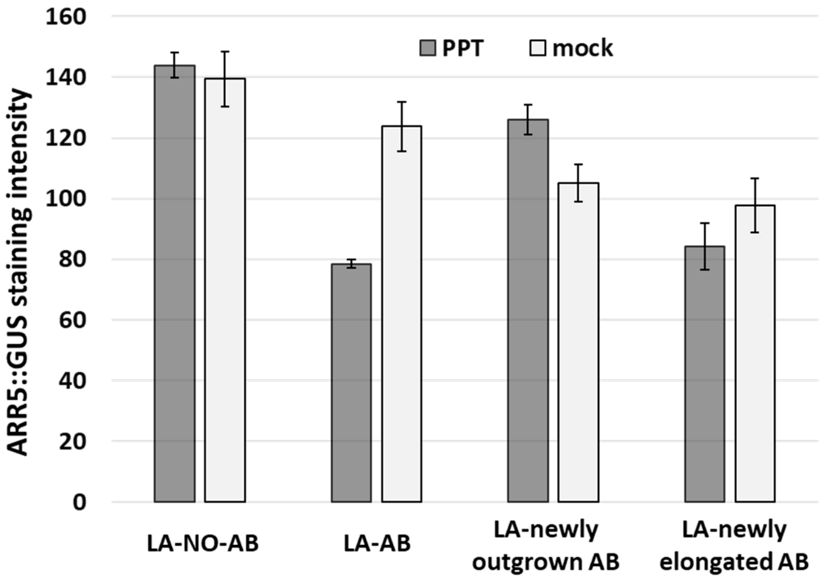


***Supplementary Figure S2.*** Measurement of GUS staining intensity in mock control and potato purple top (PPT) phytoplasma-infected ARR5::GUS cytokinin reporter tomato lines based on histogram function of Adobe Photoshop software. Intensity values were collected from the individual samples (images) according to the normalized median intensity of white background. RGB white is RGB (255,255,255); RGB black is RGB (0,0,0). The GUS staining intensity is stronger, the RGB is lower.

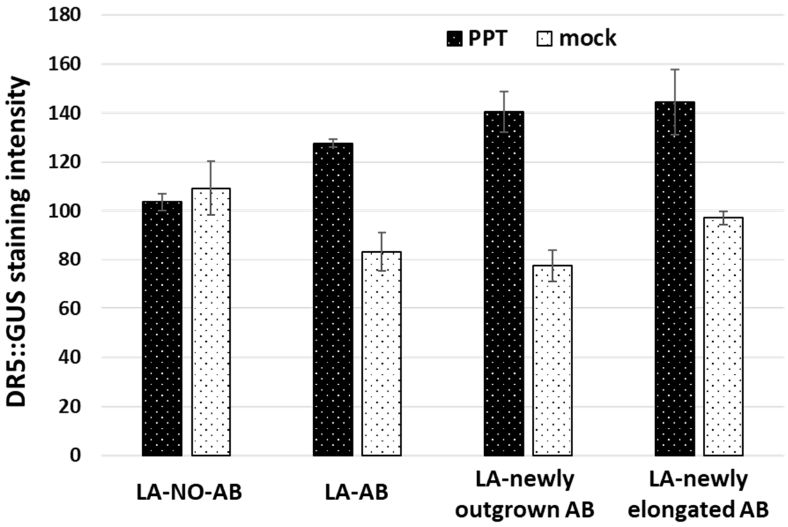


***Supplementary Figure S3.*** Measurement of GUS staining intensity in mock control and potato purple top (PPT) phytoplasma-infected DR5::GUS cytokinin reporter tomato lines based on histogram function of Adobe Photoshop software. Intensity values were collected from the individual samples (images) according to the normalized median intensity of white background. RGB white is RGB (255,255,255); RGB black is RGB (0,0,0). The GUS staining intensity is stronger, the RGB is lower.

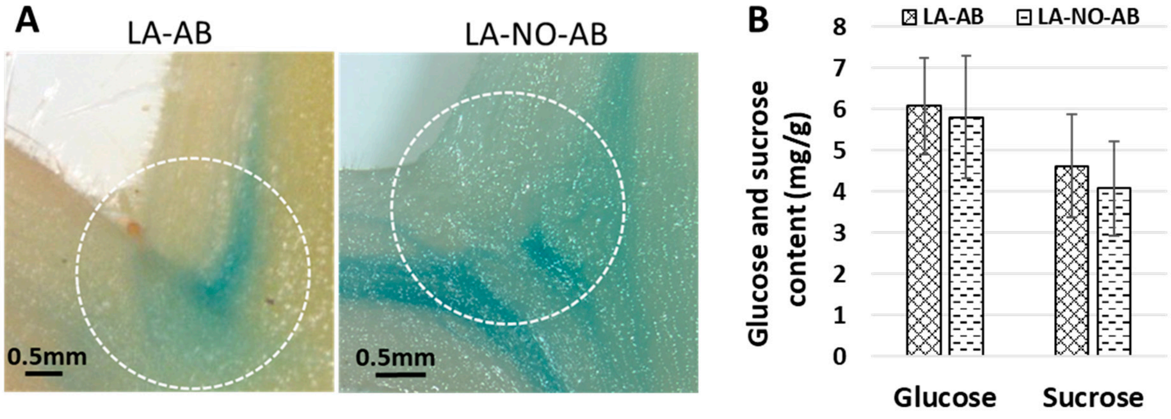


***Supplementary Figure S4.*** Distribution of cytokinin responsive signal and sugar measurement in leaf axils of ARR5::GUS cytokinin reporter tomato plants (mock controls). Cytokinin accumulation and sugar measurement in leaf axils of ARR5::GUS cytokinin reporter tomato plants (mock controls). (A) GUS staining signal (cytokinin) in leaf axil where a new axillary bud initiated, LA-AB (leaf panel) and leaf axil where no axillary bud initiated, LA-NO-AB (right panel). Leaf Axils (LA-ABs and LA-NO-ABs) with white circles were sampled for sugar content assessment. (B) Glucose and sucrose content in leaf axils (LA-ABs and LA-NO-ABs) of mock control plants.

***Supplementary Table S1.*** Length measurement of roots in mock control (MC) and potato purple top (PPT) phytoplasma-infected plants.

| **Plant** | **Length of the entire root system (cm)** | **Length of the primary root (cm)** | **Length of the longest secondary lateral root (cm)** |
|---|---|---|---|
| MC-1 | 59.8 | 5.2 | 54.8 |
| MC-2 | 44.5 | 5 | 39.5 |
| MC-3 | 47.3 | 4.2 | 44.3 |
| PPT-1 | 32.3 | 3.1 | 29.2 |
| PPT-2 | 24.5 | 3.8 | 22.3 |
| PPT-3 | 29.3 | 2.9 | 27.8 |
| MC-Mean ± SD | 50.533 ± 20.237 | 4.8 ± 1.314 | 46.2 ± 19.438 |
| PPT-Mean ± SD | 28.7 ± 9.774 | 3.267 ± 1.174 | 26.433 ± 9.061 |
| Statistical significance | $p = 0.0269$ | $p = 0.0205$ | $p = 0.0319$ |

Data were analyzed statistically with a two-sample *t*-test program online (https://www.evanmiller.org/ab-testing/t-test.html). Statistical significance <0.05. SD indicates standard deviation.

***Supplement Table S2.*** Fluorescence intensity measurement of aniline blue-stained callose in stem samples (both mock control and potato purple top [PPT] phytoplasma-infected plants) based on histogram function of Adobe Photoshop software.

| **Samples** | **Histogram readings** | **Randomly selected 10 aniline blue-stained spots** | | | | | | | | | | **Mean±SD** |
|---|---|---|---|---|---|---|---|---|---|---|---|---|
| | | **1** | **2** | **3** | **4** | **5** | **6** | **7** | **8** | **9** | **10** | |
| Mock control | Average intensity value | 91.87 | 113.56 | 59 | 52.99 | 53.63 | 84.41 | 55.1 | 47.3 | 67.12 | 36.92 | 66.19 ± 16.79 |
| | Pixel | 1256 | 206 | 696 | 359 | 1464 | 345 | 1398 | 820 | 362 | 1403 | 830.90 ± 363.04 |
| PPT-infected | Average intensity value | 140.18 | 137.59 | 146.88 | 142.53 | 147.18 | 126.73 | 161.28 | 147.33 | 114.2 | 148.47 | 141.24 ± 9.28 |
| | Pixel | 4342 | 2281 | 2548 | 2340 | 1356 | 2138 | 1145 | 2597 | 2729 | 2534 | 2401 ± 617.66 |

Data were analyzed statistically with a two-sample *t*-test program online (https://www.evanmiller.org/ab-testing/t-test.html). Statistical significance <0.001. SD indicates standard deviation.

***Supplementary Table S3.*** Primers designed for reverse transcription real-time PCR in this study.

| Arabidopsis gene<br>(Tomato ortholog, GenBank accession number) | Primer name | Primer sequence |
|---|---|---|
| asparagine synthetase<br>(*Sl-ASN*, NM_001319849) | Sl-ASN-F<br>Sl-ASN-R | TACAATCCCGCTTGGTTCTC<br>CCCATTGCTTAGCAGCTTTC |
| trehalose-6-phosphate synthase<br>(*Sl-TPS*, AB368491) | Sl-TPS-F<br>Sl-TPS-R | TGACAAACAGCAGGCTCATC<br>CTTGCTGAGCGTATCCCTTC |
| ent-kaurene synthase<br>(*Sl-KS*, NM_001321000) | Sl-KS-F<br>Sl-KS-R | CTTCCGCTACTGACGAGGAC<br>TCTCCAATCCCCTCTGACAC |
| squamosa promoter-binding proteins, SPLs<br>(*Sl-SBP1*, DQ672601) | Sl-SBP1-F<br>Sl-SBP1-R | GGGAAGGGAAGAGAAGCATT<br>GATGGTATGGCTTGGCATCT |
| BRANCHED 1, BRC1<br>(*Sl-BRC1a*, NM_001246873) | Sl-BRC1a-F<br>Sl-BRC1a-R | ACACAGCAAAATCAACACGG<br>TGCTGGCTTTATCGAACCC |
| BRANCHED 1, BRC1<br>(*Sl-BRC1b*, NM_001247643) | Sl-BRC1b-F<br>Sl-BRC1b-R | GAGTAGCAGAATTAGTGGACGG<br>GTCTCATCCTTCGATCTCTTGG |
